# No need to be rebellious: placemaking and value co-creation in the skateboarding City of Malmö

**DOI:** 10.3389/fspor.2024.1455642

**Published:** 2024-10-11

**Authors:** Karin Book

**Affiliations:** Department of Sport Sciences, Malmö University, Malmö, Sweden

**Keywords:** skateboarding, placemaking, value co-creation, urban development, participation, collaboration, space, Malmö

## Abstract

During the last 20 years, Malmö has developed into an internationally recognised skateboarding destination and a valued skateboarding place for local and regional skaters. In contrast to many cities, skateboarding and skateboarders have been appreciated and embraced in public planning and development. The aim of the paper is to discuss the development and identity of Malmö as a skateboarding city through the lens of the concepts of placemaking and value co-creation, and—not least—through the narratives of six persons with different relations to skateboarding and Malmö. The paper shows that the City of Malmö has engaged with local skateboarding communities through several initiatives that have fostered a collaborative relationship. The non-profit association Bryggeriet, a skateboard high school, an active skateboarding community, and the City of Malmö are all involved in the placemaking and co-creation of Malmö as a skateboarding city. The city's approach to skateboarding involves a bottom-up strategy, engaging non-experts in the development of spaces, reflecting a shift from traditional top-down models towards a co-creation model in order to create synergetic outcomes. Malmö's transformation into a skateboarding city is characterised by the integration of skateboarding into the urban fabric, showcasing a welcoming attitude. The paper underscores the dynamic interplay between physical, imagined, and lived spaces in urban skateboarding culture. However, it also demonstrates how a successful and professionalised model for introducing the skateboarders’ interests into the corridors of power risks undermining the link to, and the engagement of, the new generation of grassroots skaters, and maybe also the edgy, rebellious character of skateboarding.

## 1 Introduction

It is the first of June 2024. In Malmö (Sweden), people are gathering in the place behind the centrally located Johannes Church. They are skateboarding, sitting, talking, watching and waiting for the opening of LOVE Malmö—the latest skate infrastructure investment in the city. As stated by the City of Malmö (at an information sign in connection to the site):

LOVE Malmö is many things at once. It is a pedestrian walkway, a cultural monument, a stage and a lounging space. One thing it is not, however, is a skatepark. Around the world, skateboarders are offered skateparks as a means of removing them from urban spaces. LOVE Malmö invites skateboarding to be a part of the life of Malmö.

There is one particularly spectacular aspect of LOVE Malmö, namely, the fact that the place is inspired by the iconic LOVE Park in Philadelphia and has been developed as a collaboration between Philadelphia's skateboarding organisation Skate Philly and Skate Malmö. LOVE Park in Philadelphia was one of the most sacred places in the skate world. When it was demolished in 2015, the City of Malmö bought 20 tons of stone, edges and garbage cans from the site. The material was shipped across the Atlantic and LOVE Malmö is now a copy of a part of LOVE Park. By using materials from this legendary skate spot, a previously hidden part of Malmö is transformed into a lively and inclusive meeting place for everyone (1, 2). It fits well into, and adds a new dimension to, Malmö as a skateboarding city.

At the same time as the opening of LOVE Malmö, the nearby Art Centre opened a new exhibition. The main part of it consists of Koo Jeong A's skateable installation EHM (Event Horizon Malmö). In the main hall, skateboarders are invited to skate the three beautifully designed bowls. This, too, adds a new dimension to the skateboarding city: a combination of art and an indoor skatepark at an established and recognised art institution.

Outside the Art Centre, there is a square which during the last decade has turned into one of the most appreciated and well-known skate spots (referred to as “Svampen”) in the city. It was not initially built for skateboarding, but was defined by skaters as a street skate spot. In line with the Malmö approach, it is welcomed as a feature in the public space. This is probably the most essential ingredient in the skateboarding city Malmö, that is, the permissive and even welcoming approach to skateboarding more or less everywhere in the city. Some cities have started to follow Malmö's example, but the approach in Malmö is still in stark contrast to the situation in many other cities:

Although skateboarding is sometimes celebrated for its neoliberal characteristics, presently, in most contemporary cities, skateboarders’ increased exposure within plaza-like spaces still produces a lack of public understanding and leads to negative perceptions of skateboarding. This phenomenon has engendered planning responses that make skateboarding an illegal activity. Regulatory and physical barriers limit skateboarders’ use of space […] [(3), p. 464].

During the last 20 years, Malmö has developed into a recognised skateboarding destination, and a valued skateboarding place for local and regional skaters. Malmö as a skateboarding city is built around different characteristics: a skate-friendly infrastructure and a general positive attitude towards skateboarding in public space; a large array of large-scale events as well as events and activities directed to the local skateboarding community and the general public; an active, creative skateboard community and many do-it-yourself (DIY) initiatives, as well as planned efforts and support from the municipality. This is a result of consistent, constructive partnerships between the municipality and user groups. Drawing on the story of Malmö's skateboarding development as outlined by Book and Svanborg Edén (4), we can conclude that it rests on a multi-level, multi-content approach. Actors involved in the process include engaged individuals and grassroots communities as well as authorities.

Malmö's story could in many ways be considered successful, and worth analysing from different perspectives. In the paper *Malmö—The Skateboarding City: A Multi-Level Approach for Developing and Marketing a City through User-Driven Partnerships,* Book and Svanborg Edén (4) mainly examine how skateboarding as a community, a sport and a cultural phenomenon is integrated into and drives the development, branding and marketing of a city. The aim of the present paper is to discuss the development and identity of Malmö as a skateboarding city through the lens of the concepts of placemaking and value co-creation, and—not least—through the narratives of six persons with different relations to skateboarding and Malmö.

Moreover, since the publication of the paper by Book and Svanborg Edén in 2020, the context of skateboarding has partly changed, both globally and locally. Maybe Malmö has come to the end of one phase of development and now needs to find its way into the future.

The aim is not to present a thorough analysis of skateboarding in urban settings or of a revanchist city space (as this has been done in numerous publications, e.g., (3, 5–8), but to reflect on Malmö as a particular case or example, as Malmö is supposed to be well developed concerning the phenomenon that this article is focusing on. Perhaps too well developed?

With regard to terminology, when the term *City of Malmö* is used in the text, it refers to the municipality (as an administrative unit with different municipal departments). Thus, the words “city” and “municipality” are to some extent used interchangeably, whereas the term *Malmö* generally refers to the city more broadly. No distinction is made between the terms “skate” and “skateboarding”.

## 2 Theoretical approach

Since around 1980, Sweden and many other western countries have experienced a shift from an authoritarian and centrally controlled to a more communicative type of urban planning and politics (4, 9). On the one hand, citizens are given more opportunities for influence, and, on the other hand, there is the influence of commercial actors, in line with neoliberal politics (neoliberal perspectives are frequently used in discussions on skateboarding; see, e.g., (3, 10). Based on Lombard (11), we can establish a link between citizens’ engagement and neoliberalism, as people are expected to leverage their abilities and skills, act as active citizens and be entrepreneurial.

As expressed in the introduction section above, Malmö is a recognised skateboarding destination. The city is unquestionably a strong skateboarding brand that is highly valued. City marketing and branding have been *a priori*tised ingredient in the policy and development programmes of many cities. Over the decades, we have witnessed a shift in urban marketing strategies; outward-oriented image building has been supplemented with and grounded in inward-oriented identity building [for a further discussion, see, e.g., (4, 12)]. The inward-oriented dimension of city branding focuses on the residents and on local businesses and organisations. It is often stated that the branding process must be based on the local identity and on authentic values associated with the location, as artificial narratives are not effective (13). Pedeliento and Kavaratzis (14) emphasise the connection between culture, identity and image. The elitist (top-down) perspective must be complemented by a more participatory (bottom-up) approach. This goes hand in hand with the entrepreneurial, neoliberal urban governance trend presented above, but could also go against it. Aitken and Campelo (15) highlight the importance of co-creation in branding as an open-ended process, “so that meaning is achieved through the interplay between social relationships and communal experience” (p. 916). Co-creation changes the control and ownership from belonging to the firm (or in this case the city) to being consumer-centred, where rights, roles, responsibilities and relationships might be altered and intertwined. We have witnessed not only a shift from outward-oriented to more inward-oriented city branding, but also an increasing interest in speaking about placemaking rather than place branding. Placemaking is a complex concept and process, which will be elaborated on below. Let us start with a selection of similar, but not identical, definitions of placemaking:

• According to the American non-profit organisation Project for Public Spaces, “placemaking refers to a collaborative process by which we can shape our public realm in order to maximize shared value” (16).
• The Swedish National Board of Housing, Building and Planning (Boverket) defines placemaking as: “a method for place development that combines physical place building and the creation of place relationships. The method aims at relationships between people, as well as relationships between people and places” (17).
• The Australian knowledge and education collaboration Placemaking.Education presents the following definition: “Placemaking is a philosophy and an iterative, collaborative process for creating public spaces that people love and feel connected to” (18).
• In the words of the EU-funded COST action Dynamics of Placemaking, “placemaking is […] understood as the place-related identity of the urban citizens and their collective re-imagination and reinvention of the spaces (19), the understanding of knowledge production includes all forms of citizens” knowledge connected to place and placemaking’ (20).

As seen in these examples, placemaking could be viewed as a process, a method and a philosophy concerned with using relations and collaborating with citizens in re-inventing and developing (public) places and creating shared value. Obviously, one distinct definition does not exist. Even the spelling can vary. As Lew [(21), p. 449] puts it: “Place making”, “place-making” and “placemaking” are three ways of spelling a popular concept that has at least two broad definitions in the academic literature, as well as many finer definitions’.

Spelling as such might be a minor issue, but Lew (21) uses the different spellings for labelling different interpretations. He uses “placemaking” when referring to more deliberate and purposeful top-down approaches to place creation, and “place-making” when referring to more spontaneous and unstructured bottom-up approaches. The latter is often more fundamental to the cultural soul of a place, and developed in an incremental manner, frequently driven through individual agency. Urban development and planning strategies attempt to facilitate organic place-making by inviting local or indigenous influence over development decisions through public participation and community-led initiatives. Dash and Thilagam (22) conclude that the process of placemaking “is accomplished by not only viewing place as a static spatial aspect and designing the physical form, but also by taking into consideration the social processes that shape and construct cities” (p. 545).

Rachel et al. (23) highlight the relationship between top-down strategies and bottom-up tactics in urban placemaking, and identify three key processes for urban placemaking projects. The first is *tactics*, referring to “bottom-up activities which take advantage of local conditions, engage local communities, undertake direct action, and construct a collective vision for the project” (p. 503). The second is *manoeuvres* “in which tactics are undertaken strategically, and strategies are exploited tactically to negotiate and exploit formal processes to unlock the formal systems necessary to realise the project” (p. 503). The third is *strategies*, meaning “top-down strategies that affect the realisation of the project in positive ways (such as grants to support community-led initiative) and restrictive ways (such as legal contexts and land ownership) that can work either to advantage or disadvantage the project” (p. 503).

Collaborative and participatory processes are essential in the place development process and could break down the boundaries between experts and non-experts. In traditional urban development processes, the planners (or similar) are considered to be the experts who take the lead and control the processes. In participatory urban processes, “non-experts” become part of the development team. With a reference to, for example, Robertson and Simonsen (24), Rachel et al. [(23), p. 495] underline that non-experts are in fact experts: “As users are experts of their own lived experiences, they can participate in design decisions and even participate as designers themselves”. Another researcher, Richelieu (25), emphasises co-creation and cooperation involving multiple stakeholders, and the importance of “willingness from managers and politicians to communicate, collaborate and find a consensus for the benefit of the local population” (p. 366).

As mentioned, in this paper the placemaking concept will be linked to value co-creation. The conceptual approach of value co-creation originates from the disciplines of marketing and service sciences (26). Value co-creation is, as discussed by Janinan et al. (27), a step-wise process with, and for, multiple stakeholders, through regular, ongoing interactions leading to innovation, increased productivity and co-created outcomes of value. Woratschek et al. [(28), p.12] apply a more commercial perspective on value co-creation:

[….] value is co-created in a collaborative process between firms, customers and other stakeholders. Actors (e.g., firms, customers, non-profit organisations and government) actively participate in the value co-creation process by integrating resources from (one or more) service providers with their personal (e.g., knowledge, competencies and skills) and other resources.

Often, value co-creation concerns companies and customers. Vargo et al. (29) highlight that the actors (both the company and the customer) employ and share their competencies, in terms of, for example, skills and knowledge, to foster value creation. Although value co-creation is often used in commercial contexts, there are examples of other contexts as well. For example, Toukola et al. (26) focus on the co-creation of value by coalitions of public and private actors in urban development projects. In their study, they ‘rely on the assumption that the municipality's and end-user's value correlates because the municipality can—and to some extent, it must—take into account the end users’ (i.e., citizens) perspectives by engaging them’ (p. 4).

Both placemaking and value co-creation are based on the collaboration with and the participation of different stakeholders. Arnstein (30) illustrates eight levels of involvement and power through the ladder of participation. Participation in practice is often carried out in terms of legitimisation, information, manipulation and tokenism—that is, the lower levels of the ladder. Only steps six to eight concern real citizen influence: different forms of partnership are developed, a certain amount of control and power is handed over, or complete control over the processes and their results is given to the citizens (30). According to Rachel et al. [(23), p. 495], citizen control reflects some of the tactics of participatory placemaking.

To sum up, this paper uses the concepts of placemaking and value co-creation as a conceptual framework when discussing Malmö as a skateboarding city. Here, the value co-creation involves the municipality and the users/skateboarders (i.e., collaborations between public and mainly non-profit organisations), and is defined as a process in which stakeholders collaborate with and influence each other to create opportunities for synergistic outcomes. As stated by Savage et al. (31), collaboration could enable organisations to achieve results or find solutions they could not achieve on their own.

Also, when using the concept of placemaking, the paper is inspired by Richards (32), who relates placemaking to Lefebvre's triad [see (33)] of physical, imagined and lived space, “where physical space is formed of materials or resources, the imagined space gives and takes meaning from the social and cultural context and the lived space is the recursive result of the creativity of users of the space” (p. 10). The physical space is experienced and transformed by users through their everyday lives. In relation to this, Richard refers to Soja (34) and the concept of “thirdspace”. Soja's concepts “firstspace” and “secondspace” consist of the “real” (physical, built) and the imagined, representational space. Thirdspace combines first- and secondspace in “a fully *lived space*, a simultaneously real-and-imagined, actual-and-virtual, locus of structured individual and collective experience and agency” [(35), p. 11]. With inspiration from Soja (34), Shove et al. (36) and Richards (37), Richards [(32), p. 11] presents a framework of three spaces describing the relationship between place and placemaking practice:

• Physical space: Materials—buildings, urban form, design, public space, etc.
• Imagined/symbolic space: Meaning—symbols, icons, identity, narrative, storytelling
• Lived experience: Creativity—patterns of daily life, routines, tempo

## 3 Method

To give a background to the skateboarding city Malmö, material from the previous paper by Book and Svanborg Edén (4) is used, supplemented by other material (e.g., news articles). The background, which is presented in section 4, is related to the theoretical framework, that is, the concepts of placemaking and value co-creation.

In Section 5, the discussion on the development and identity of the skateboarding city Malmö is built on interviews with six persons involved in or affected by the skateboarding identity and the making of Malmö as a skateboarding city. Those six persons are: the official skateboarding coordinator at the City of Malmö; two civil servants at the Business Department of the City of Malmö; a manager of a skateboarding association in Malmö; a former Malmö skateboarder and resident; and a skateboarding researcher in San Diego who visits Malmö on a regular basis. Hence, the informants represent different sectors with inside as well as outside perspectives, and with different interests in and relations to skateboarding. Despite their different perspectives, interests and experiences, I would define all of them as stakeholders in relation to Malmö as a skateboarding city.

Four of the informants are experienced skateboarders, while two are not skateboarders. Three of them are women and three are men. Except for one, the informants are older than 40. In this study, adolescents are not represented, the focus being on the perspectives of adult experts. Focusing instead on the new and future generations of skateboarders in Malmö, would be a relevant next research step.

The interviews had a semi-structured format and revolved around questions regarding the informants’ relation to Malmö as a skateboarding city, the perception of Malmö as a skateboarding city and its identity as such, values, placemaking and the past vs. the present. The interviews were conducted in the period February to May 2024. Each interview lasted 55–90 min. Three interviews (with four informants, as two informants representing the same department were interviewed together) took place either in the workplace of the informant/s or at a neutral place selected by the informant, and two interviews were conducted on Zoom. All interviews were recorded and transcribed. The interviews were then analysed through a content analysis, and the results are presented mainly in the form of quotes.

The informants are listed below, with correct first names but no surnames. None of the informants have requested anonymity.

• Anna: civil servant and head of the unit Marketing and attractiveness, at the Business Department, City of Malmö.
• Ulrika: civil servant and head of the unit Innovation and development, at the Business Department, City of Malmö.
• Gustav: civil servant and skateboarding coordinator at the Streets, Parks and Property Department, City of Malmö.
• Nils: operational manager at the non-profit skateboarding organisation Bryggeriet, Malmö.
• Neftalie: PhD, assistant professor, skateboarding researcher, at San Diego State University, USA.
• Emma: former Swedish skateboarding champion, former Malmö resident, former representative in the Swedish Skateboarding Federation.

The author of this article has conducted research on skateboarding before, but is not a skateboarder and hence not an insider in the skateboarding community.

## 4 Malmö as a skateboarding city: a background

Malmö is a medium-sized, former industrial city in the south of Sweden. The city has a very diverse and young population and a marked socioeconomic polarisation. In order to transform into a modern, post-industrial city, Malmö has, since the 1990s, on the one hand launched a politics focusing on city branding with neoliberal undertones (38, 39), and, on the other hand, also acknowledged its urban challenges and partly problematic image. It has started to develop a large variety of strategies to improve the living conditions for its residents. Among those strategies, we find support for urban sport, development of public spaces and grassroots initiatives. On the city's webpage (40) under the heading “The story of Malmö”, it says: “How a post-industrial city reinvented itself as a dynamic knowledge centre built on cultural diversity, youth and sustainable development”. Today, ideas of participation, diversity and youth are celebrated in words, in practice and in the branding of Malmö. The development of skateboarding is one example of this.

The development of Malmö into a recognised skateboarding city and destination illustrates how consistent, constructive collaborations between the municipality and user groups can generate placemaking, place development and place branding. Below, a brief outline of Malmö's development as a skateboarding city follows, mainly building on Book and Svanborg's thorough resumé of the development.

The skateboarding story started in the 1990s. At that time there were no skateparks and the skateboarders skated in a parking garage underneath a centrally located shopping centre, causing annoyance and conflicts. Local youth organisations set out to help the skaters communicate with the municipal authorities. In 1998 this resulted in the establishment of a not-for-profit association called Bryggeriet and an indoor skatepark with the same name (meaning “the brewery”, as it is located in a former brewery). Bryggeriet became an institutional platform for community-based initiatives. The skateboarders’ entrepreneurship coupled with municipal support allowed Malmö's skate activities to grow. This constituted the starting point for the making of the skateboarding city.

### 4.1 Skateparks and events

An early initiative was the skateboarders’ own project plan for a concrete skatepark. The idea coincided with municipal objectives to establish public space for youth within the ongoing redevelopment of the harbour area. Therefore, the plan for the skatepark (named Stapelbäddsparken, which refers to the location in the old shipyard) gained attention and was approved. The skaters convinced the city to use skater-run construction companies and let the skaters of the city participate in the building themselves. The result was one of Europe's largest and best concrete skateparks, opening in 2005. It was also the apprenticeship for Malmö's own skatepark construction industry. Hence, physical or material value, knowledge and empowerment were co-created.

Having a modern skatepark, Bryggeriet reached out to the brand Quicksilver, offering to co-host their European Championship event Bowlriders. The event required support from the municipality's Streets, Parks and Property Department, building on the already established collaboration between Bryggeriet and the municipality. Bowlriders introduced large-scale skateboarding events to the general public in Malmö, and gained local and international exposure. It also established a recurring municipal budget for skateboarding activities, which enabled Malmö to launch the event brand Ultrabowl when Bowlriders was discontinued.

Bowlriders was the first event in a row of international skate events to be hosted in Malmö. For instance, when Vans Park Series launched their first global World Championship tour in 2016, Malmö was selected as the partner city. The well-established municipal partnership acted as a guarantor and Malmö was able to influence the event to include the establishment of a permanent legacy skatepark and a female competition. The event significantly increased the exposure of Malmö as a skateboarding city, and the legacy park both generated long-term material and social benefits and set the stage for future championships in 2017 and 2018. Park Series also provided a stage for local Malmö talents, including Oskar ‘Oski’ Rozenberg-Hallberg, who claimed the World Championship title in 2017 and in 2019. To conclude, the collaboration and knowhow developed when planning and building Stapelbäddsparken, were used and increased in a spiralling way. It aimed to attract and host events, and to improve the conditions for not least the formalised and professionalised skateboarding, as well as building an urban skateboarding image and identity. This would not have been possible without the combination of bottom-up initiatives and insights from the skateboarding community and top-down support and power tools at the municipality. From a placemaking perspective, it could be viewed as a “top-tier” or high-profile type of development of a place, focusing on physical space but having long-term effects on symbolic and lived space as well.

### 4.2 Organisational setting

As mentioned above, the establishment of the not-for-profit organisation Bryggeriet in 1998 laid the foundation for the development of the skateboarding city. Besides being involved in the international events, Bryggeriet also developed community-oriented events in order to hold on to the grassroots and cultural credibility. Another important milestone was the establishment of Bryggeriet High School in 2006, creating the opportunity to combine skateboarding with formal education. It stands as a key element in Malmö's skate identity and is a platform, a magnet and a forum for skaters from across Scandinavia for participating in and help build Malmö's skate scene.

The construction of the skatepark and Malmö's recurring skateboarding events developed the relationship between the Streets, Parks and Property Department (City of Malmö) and the association Bryggeriet. In 2012, this was formalised under the brand name and communication platform *Skate Malmö*. To further formalise the skateboarding efforts, in 2015 a full-time municipal civil servant, recognised as the skateboarding coordinator, was employed at the City of Malmö.

The skateboarding coordinator's position within the municipality opened opportunities to move the skateboarding activities out of the skateparks into public space. Having an initiated person from the skateboarding community within the municipality administration is to integrate the grassroots perspectives into the formal development of the city. For instance, the event concept Skate Malmö Street (SMS) was developed to extend the social impact of events by introducing permanent skateboarding infrastructure in existing urban spaces. SMS also set out to promote female and non-binary skateboarding. In 2019, the fusion of events and urban development motivated the partnership with the London-based Pushing Boarders conference and the “Dork Zone” project, a strategy for introducing skateboarding into new neighbourhoods through placemaking and community building methods.

To sum up, there are a number of stakeholders involved in co-creating the skateboard product of Malmö as a skateboarding city: the not-for-profit organisation Bryggeriet (which is a platform and a strong voice for the skaters in Malmö), a skateboard high school, the City of Malmö and an active skateboarding community.

### 4.3 Public spaces and DIY

At the Pushing Boarder conference in 2018, Iain Borden, one of the most influential researchers on the practice of skateboarding in urban space, described Malmö as ‘the most progressive city in the world for skateboarding. It's managed to embrace, integrate and manage skating in its urban fabric’ (41). The development of skateparks, described above, forms the formal skating space in Malmö. However, that is probably not what Borden is referring to. Rather, Malmö's image as a skateboarding city is based on the municipality's willingness to integrate skateboarding as a welcomed element in public space, partly because the skateboarders have a voice through the skateboarding coordinator inside the corridors of the City of Malmö. In an interview about urban pioneers made by Euro Cities (42), he states:

For example, when we adjust public spaces for skateboarding, we also make sure the skateable infrastructure does not communicate that it is exclusively made for skateboarding but for other functions too. We have introduced skateable furniture, sculptures that function as spaces to rest or abstract objects for kids to play. What we are trying to achieve are spaces that are open to engagement, play and interaction between different urban users.

Malmö's skateboarding events and parks have received significant public investment. They constitute the formal façade of skateboarding. Moreover, the skate-friendly attitude in public space has created a strong skateboarding identity and been a source of inspiration to many other cities [see, e.g., (43)]. To dedicated skateboarders, however, Malmö's do-it-yourself (DIY) history is of greater significance than any championship event is ever likely to be. Inspired by skaters in Portland, Oregon, Malmö skaters began claiming unused urban spaces to build their own skate spots in concrete. Gathering to build these spots contributed to solidifying the community and shaped parts of the identity of the Malmö skate scene (6). These largely illegal DIY projects helped connect the Malmö skaters to Portland-based skatepark builders, frame the idea of Stapelbäddsparken and lay the foundation for Malmö's own skatepark building industry.

Malmö's DIY scene inspired a path to self-determination through skaters building their own spots and community, and it attracted the attention of photographers and filmmakers (e.g., Pontus Alv, who made *The Strongest of the Strange* and *In Search of the Miraculous)*, and of international skateboard media, generating numerous documentaries and articles. The Malmö story gained importance as a reference in a cultural progression where skaters around the world began initiating their own DIY projects (6).

This has generated valuable exposure for Malmö, and, more importantly, placemaking and value creation grounded at the grassroots level, driven by storytelling. Informal storytelling has become an ever-growing, integrated part of skateboarding (44). Connecting to the concepts of the spatial triad, DIY could represent not only the physical but also the symbolic and lived space, from a skateboarding community perspective, or it could be said to represent thirdspace, using Soja's concept.

### 4.4 Skateboarding undergoing change

The story about the skateboarding city is in many ways a successful example of stakeholder collaboration, value co-creation and placemaking. However, during the last five to ten years skateboarding has moved into a new phase both internationally and in Sweden. The commercialisation, professionalisation and formalisation processes have continued.

Taking off in the 1990s, commercialisation is not new in skateboarding. On the one hand, there is a growing market for skate products and, as a consequence, a growing number of skateboard-related companies. Malmö has attracted and been a breeding ground for numerous businesses. On the other hand, the skateboarding event sector has undergone both growth and commercialisation, which has, in turn, pushed professionalisation and attracted a wide array of sponsors also from outside the skateboarding community.

Skateboarding moved further towards sportification [on sportification of skateboarding, see (45, 46)], when becoming an Olympic sport added to the 2020 Olympic programme (postponed to 2021 due to COVID-19). Schwier and Kilberth (47) discuss if this means that skateboarding as a subculture is about to lose its identity, when moving from play to sport and into podiums. World Skate is now the international governing body in the world for all sports performed on skating wheels (48). This is yet another step towards further formalisation of skateboarding.

As underlined by Bäckström and Blackman [(49), p. 121], “Skateboarding has evolved from a creative urban activity with a legendary past meshed with subcultural values into an Olympic sport and a platform for multinational industry and global enterprises”. Skateboarding thus has several different, but partly overlapping, faces.

The skate scene in Malmö has been affected by the above trends and processes. Bäckström and Blackman (49) raise the question whether skateboarding has become mature and mainstream. In the case of Malmö, a level of consolidation has in one way been reached, while the new influences and trends mentioned above have at the same time altered the scene.

## 5 Malmö as a skateboarding city: the interpretation of six stakeholders

### 5.1 Key elements of placemaking

In the interviews, the informants were asked to elaborate on their perceptions of placemaking in Malmö in relation to skateboarding. They highlighted slightly different things.

When introduced during the interview to the spatial triad developed by Lefebvre and Soja, the skateboarding researcher from San Diego, Neftalie, defines three types of physical space characteristics. In Neftalie's words, these are regular spaces, skateable spaces and shared spaces. By regular spaces Neftalie means formal structures, like the skateparks. Skateable spaces are spaces not primarily developed for skating, but equipped and prepared in a way to suit skateboarders. Shared spaces are all those spaces where skateboarders appear together with other users.

Neftalie underlines the importance of Malmö's welcoming attitude towards skateboarding in public spaces. He finds this welcoming attitude unusual, yet in line with the international, urban discourse problematising the privatisation of public space in many cities. Malmö shows that it is possible to have truly public and more inclusive spaces. He reflects on how he has perceived the public space in Malmö, with regard to shareable and shared spaces, when visiting and skating here. Many of the public spaces in Malmö encourage multiple purposes and the integration of different uses and users, and Neftalie discusses the opportunities of public space in Malmö in terms of “rethinking the relationship with each other”, the importance of “recognis[ing] others than yourself” and the potential of “intergenerational learning and connection”. He says: “We see each other if we are in the same space”.

According to Neftalie, it is also important to allow people to interpret the places themselves. He thinks that Malmö is good at repurposing places. Maybe, repurposing spaces is a way of remaking physical spaces adapted to lived spaces.

Nils, the operational manager at the non-profit skateboarding organisation Bryggeriet, also mentions the places as central in the placemaking, and specifically emphasises the importance of the DIY sites. In fact, he calls the DIY places ‘the ultimate artefact for placemaking in the skate city’. Moreover, they are unique, come from the grassroots and are the main reason for putting Malmö on the map, he claims. Thus, an interpretation of what Nils says is that the DIYs are physical places formed by lived experience and loaded with symbolic meaning.

Nils says that from the beginning the DIY places went under the radar. After the construction of Stapelbäddsparken, the city's interest in the DIY sites increased and they were seen as okay by the municipality. They have, Nils asserts, great value in the skate world and have gained exposure, for instance, in Pontus Alv's films. Moreover, Nils identifies skate events as a breeding ground for placemaking and value co-creation, events being something concrete to gather around and with different groups and competencies being invited in the process.

Emma, the former Swedish skateboarding champion, Malmö resident and representative in the Swedish Skateboarding Federation, lists a number of things that, according to her, constitute the material or physical space of the skateboarding city Malmö: skateparks, Bryggeriet, the companies around skateboarding and good transport connections.

“Originally”, she says, “Malmö was really a poor skate town. But they did their own thing and developed the city”. So, in fact, turning a place into a skateboarding city seems to be about placemaking instead of favourable basic conditions. What Emma says can be connected to Lefebvre's original concept of the representational space. Regarding placemaking, Emma states: “When you're in it, you just do it, but when you look back, you realise how crazy it is!” She starts enumerating a number of values that were created in the making of the skateboarding city—values for the individual, like belonging and community—emphasising that these are not always prioritised in today's society. She goes on to talk about female empowerment; the corporate development; her own events and competitions; cultural exchange, when people move to Malmö; monetary value; and a cool image. Emma also underlines the diversity in actors involved in the making. Especially, all those who filmed and took photos were important in the process, and not least the non-profit association Bryggeriet was involved and created a lot. Emma then talks about the girls’ skater group Tösabidarna; the highschool Bryggeriet; the skate store Street-lab and Pontus Alv and his company. What she does not mention spontaneously is the City of Malmö. When asked about it, she says: “It hasn't affected me that much, their activities are too planned. I just wanted to operate at the grassroots level. To me, the City of Malmö is a sack of money and has no other meaning for me”. However, she admits that the efforts of the municipality have borne fruit.

Gustav at the Streets, Parks and Property Department, reflects on the fact that the different spaces are intertwined and connected. Imagined space is not only created by planners and urban developers, but also by the skate community, through posting films, images, etc. This is partly based on the physical space and all the investments that have been made, not only materially but also in the form of other structures (like networks), and on the attitude towards skate that was held in Malmö. Lived space does not always correspond to imagined space, but in Malmö there is an opportunity to influence the physical space through the lived experiences, Gustav thinks.

The civil servants (Anna and Ulrika) at the Business Department focus less on the places and instead more on the branding and image aspects. They stress the role of skateboarding for the place brand, and for a dynamic and renowned community. As Ulrika says,“People come here because we are strong and well-known in skate”, and “it's part of Malmö's DNA”.

### 5.2 The skateboarding city today

In the interviews, we also talked about where Malmö stands today, after two decades of world-class skateboarding development.

Neftalie reflects from an international perspective and seems to see Malmö as a role model. Malmö's position has given rise to valuable exchanges and cooperations with cities around the world. For example, in the HYBRID project, Malmö worked together with Bordeaux and Toronto with skateable sculptures in the urban space. Furthermore, he points out that Malmö's way of working with skate has raised ambassadors of the skateboarding city. Nils as well thinks that Malmö's skaters are proud and find it fun when Malmö is shown off: “What we created together, the collaborations, the study visits and the unique environment.”

Nils is also a bit self-critical. “We have tried to do everything”, he says. “This means jumping from chair to chair, leaving empty chairs behind. Now, we need to stop and develop more awareness”. Moreover, he mentions that Skate Malmö, the collaboration platform, has had an important function. However, the high ambition that Skate Malmö represents (in the form of skating scene, events, groups) is difficult to maintain in the long run. “It's a lot of work.” As a consequence, the ambitions have been lowered and the focus is now on the joint events and on building around them. Nils sums up: “Now we are at the point where the meaning of Skate Malmö is perhaps just confusing. It's easier to handle it separately. The idea is good but hard to sustain. However, it has inspired other cities.”

Emma starts by reflecting about the skateboarding city when she was young, some ten to fifteen years ago. She emphasises that it was incredibly creative, that there was no pressure, and that there was a focus on building up something new. It has partly changed: “When the Olympics came in, it's not the same kind of people anymore. It's more established. Even DIY initiatives are funded and sanctioned. It's market-driven, due to the Olympics, companies, sponsors, competition, elite investments—a different skate scene. But still, the other skate community remains”. She says that the two communities or scenes meet at Bryggeriet in winter, when they need to be indoors. The moderate size of Malmö and the lack of hierarchies promote the meeting and the co-existence of the different parts of Malmö. When asked if the city has become too professional about skateboarding, she says that it is not the same scene anymore, but that it is a natural development taking place. “Standing still can really kill a scene.” According to Emma, Malmö is still a relevant skate city and it is the strength of Malmö to keep up with development. However, she sees a problem if the marketing does not feel genuine and if big brands take over: “Malmö city cares about Malmö, Nike doesn't”.

One of the two civil servants at the Business Department in Malmö can identify a slight shift in the role of skateboarding in the urban politics. “Skateboarding isn't as hyped as ten years ago”, she maintains. Then, it was a separate and outstanding phenomenon, but now it is an integrated phenomenon that is not highlighted separately. Today, they rarely talk explicitly about skate, as they did in the past. However, the two informants elaborate on the possibilities that the skateboarding efforts have contributed to how urban development is handled, through spill-over effects to other sectors. The City of Malmö employ a wide array of collaborative placemaking methods and talk in terms of co-creation, re-doing, re-thinking and the third place. They seem to have developed a method, or almost a business model, grounded in the humus at the grassroots level. Ulrika refers to the brand of Malmö as “variegated, creative and unpretentious”. Here, we look back at the skateboard brand, and the civil servants say that it fits very well with Malmö. In Ulrika's words, “the skate image perhaps embodies Malmö or the self-image as the cool underdog”. However, then Anna adds that there is a problem: the white, middle-class dominance in skateboarding stands in contrast to the multi-ethnical, socially mixed city.

Neftalie, who is very interested in skateboarding from perspectives of diversity and ethnicity, talks of Malmö's skateboarding efforts from a learning perspective. Those efforts create opportunities for reflecting on what shared spaces are. Moreover, Neftalie believes that we can draw conclusions on how skateboarding can be used for solving other issues in society.

In relation to the issue of inclusion, Nils talks about some recent projects aiming to introduce skateboarding and skate culture in the socio-economically weaker, ethnically mixed suburbs. These are collaborative projects between the association Bryggeriet and the City of Malmö, but they also need anchoring at the local youth centres. A problem has been an unclear division of responsibilities because different municipal departments have different areas of responsibility. Also, these projects are more of top-down initiatives.

Nils has a long history in the development of Malmö as a skateboarding city, as he was one of the persons who established the association Bryggeriet in the mid-1990s. He talks about the incredible journey from zero to where we are today. The group who embarked on this journey had the same starting point: they were skaters. “Then we have developed the product in different directions as school leaders, association developers, civil servants at the municipality, filmmakers, brand developers, and so on”. They all started at the grassroots level, but then became part of the professionalisation of Malmö as a skateboarding product.

Both Gustav and Nils reflect on the driving force at the grassroots level today. Now, it is all handed to you on a silver platter and it does not require that much commitment. When they started there was nothing. As Gustav describes it:

Fighting for one’s cause, getting involved, being involved in planning and feeling anticipation are important driving forces. These often disappear when it [i.e., the project, place, etc.] is finished. How can you create continued commitment? Perhaps by not providing ready-made infrastructure, but enabling continued development, creating flexibility, and causing new goals to be set.

Then a natural question would be: Can the skaters themselves not carry on the work now that the municipality has helped and supported for this long? Gustav's response to this is: “Well, in part it would be possible. However, what would likely happen is that they would be forced to turn to commercial actors and then the holistic perspective would be lost”.

### 5.3 What about LOVE Malmö?

Let us end where this paper started, with LOVE Malmö.

The skateboarding coordinator Gustav says that “after the pandemic, the administration gave their approval to proceeding with LOVE Park from Philadelphia. Malmö's investment in LOVE Park is about showing that skating has a place in the city centre, unlike Philadelphia. It will be interesting to see how the media receives the LOVE Park investment”.

Nils at Bryggeriet highlights the uniqueness of the LOVE Malmö project. “The skate world can't believe we're doing it! However, it's a generational thing. The older skaters are lyrical, but maybe the younger ones don't understand the greatness. We'll have to make them understand. We are working with an exhibition, films, and so on”.

The American researcher Neftalie talks of LOVE Park in Malmö as city diplomacy, once again referring to international, but also local, relations.

However, only one of the two civil servants at the Business Department in Malmö knew about the project LOVE Malmö. She expressed some scepticism, without specifying why.

In relation to this, it has to be mentioned that the chairman (the leading politician) of the technical committee in the City of Malmö, gave a speech during the opening of LOVE Malmö. He seemed very proud of the project, highlighting that in Malmö, we do it differently. He ended his speech with the words “Malmö is not a city; it is an attitude”.

LOVE Malmö is at once a first, second and third space, incorporating the three dimensions of the triad [see (33)] of physical, imagined and lived space, “where physical space is formed of materials or resources, the imagined space gives and takes meaning from the social and cultural context and the lived space is the recursive result of the creativity of users of the space” [(32), p. 10]. It is a physical place, full of symbolic meaning and truly iconic, now transforming into a shared public space of different uses and with different users.

Is LOVE Malmö a step into the future? It has taken the skateboarding city Malmö to a new level, especially in terms of uniqueness and international reputation. But, as stressed by Nils, it is mainly of interest for the older generation of skaters.

### 5.4 Summarised reflections from the interviews

The different roles of the informants are reflected in what they emphasise in the interviews.

The skateboarding researcher (and skateboarder) from the USA (Neftalie), primarily focuses on the outcomes (values) of placemaking in the form of spaces (including the relation to space) and international relations. Malmö clearly has a very high international status when it comes to placemaking from a skateboarding perspective. When looking ahead, he sees the potential of using the skateboarding development for understanding and developing other sectors and phenomena.

The former skateboarding champion from Malmö (Emma) has a grassroots and skateboarding community perspective, highlighting the creation of the skateboarding city through places, groups, events, the culture and the representation in films and pictures. She recognises the efforts made by the City of Malmö, but seems to make a clear distinction between the community and the municipality (although the skateboarding coordinator is mentioned in positive terms).

The two representatives for the formal facilitation of skateboarding in Malmö—Nils at the non-profit association Bryggeriet and Gustav at the Parks, Streets and Property Department—both have a more professional and comprehensive understanding of how different organisations, stakeholders, collaborations, attitudes, etc., jointly have underpinned the placemaking and value creation. However, they also express an understanding of the fact that the high degree of professionalisation and formalisation could be a weakness when it comes to connections with the new grassroots and the ability to sustain the high ambitions of for example collaborations.

Finally, the civil servants at the Business Department mainly focus on the image value, but also on the fact that skateboarding has become an integrated part of the urban development and processes.

## 6 Discussion

When studying the development of Malmö as a skateboarding city from a placemaking and value co-creation perspective, different phases appear. If we start by looking back a few decades, the first phase was *the progressive development phase*. Malmö developed into a skateboarding city built around different characteristics, as mentioned earlier: a skate-friendly infrastructure and a general positive attitude towards skateboarding in public space; large-scale events as well as events and activities directed to the local skateboarding community and the general public; an active, creative skateboard community and many do-it-yourself (DIY) initiatives, as well as planned efforts and support from the municipality; and a large number of actors in different sectors, at different levels, engaging in skateboarding in different ways and co-operating. Thus, Malmö as a skateboarding city developed both in an organic way, by allowing and integrating new ideas and initiatives along the way, and in a structured, conscious way through a well-established organisation of power. Internationally, the development of skateboarding in urban contexts is frequently discussed from a neoliberal perspective [see, for example, (3, 10, 50)], where skateboarding is framed in the intersection and negotiations between the skateboarders’ activism or entrepreneurialism, the public good and the profit-making sector. No doubt, neoliberal forces have been in play in Malmö as well. However, the Malmö approach is more than a neoliberal strategy. It is far more progressive and comprehensive, focusing on public and non-profit collaborations rather than profit-making.

This paper emphasises the significance of DIY (do-it-yourself) sites in placemaking. These sites are unique, grassroots-driven and have played a major role in developing Malmö as a recognised skateboarding city. Connecting to Arnstein's ladder of participation in relation to placemaking, Rachel et al. (23) mention DIY urbanism as an example of the higher levels of the ladder in terms of direct citizen actions. Through the DIY actions and an active re-definition of public space, young people become “active agents” in urban development. In Malmö, this has been embraced rather than resisted.

Skateboarders’ ability to appropriate, contest and use space differently than other citizens has been illustrated in several studies [e.g., (5, 50)]. Skateboarders have a special eye on the street and urban landscape, and hence have the potential to contribute to the production, reproduction and regeneration of urban space—or even be ”shock troops” of gentrification [(51), p. 40]. In more and more cities skateboarding is viewed as an attractive urban force and a desirable part of public life, which Howell noted already in 2005. However, the extent to which the official urban developers (i.e., those who possess power) recognise the potential of skateboarders, varies. So does, not least, the degree to which the skateboarders are invited as active agents in the urban development.

McCormack and Clayton (9) discuss young persons’, and more specifically skateboarders’, influence on local policy, in terms of, for example, social and cultural capital, as well as bridging. When looking back on the development of Malmö as a skateboarding city, the capital of skateboarders has been valued, and, in return, capital for participating in formal urban development processes has been developed among skateboarders. Book and Svanborg Edén (4) highlighted that “Malmö's example suggests that engaging with these user-groups can favourably be done with respect for cultural integrity and expertise. Municipalities can do well in trusting young people to define what is cool and deploy strategies enabling them to do more of it” (p. 176).

So, Malmö has established itself nationally as well as internationally as an outstanding skateboarding city, developed initially through bottom-up initiatives followed by grounded top-down strategies. As described in the theoretical approach above, Rachel el al. (23) identify three key processes for urban placemaking projects: from bottom-up *tactics*, through *manoeuvres*, to top-down *strategies*. In Malmö, the development of the skateboarding city moved fast from tactics to strategies. In the process, the links to the grassroots were tight, partly because the people at the strategic level were grounded in the local skateboarding community and culture. They brought the social and cultural capital of skateboarding into the corridors of power and helped bridge between different sectors and levels. As representatives of the skateboarding community, they created legitimacy both at the top and at the grassroots level.

During the last years, a new phase has been entered: *the professional maturity phase*. Not only has skateboarding been increasingly formalised and professionalised as a sport, but the planning of skateboarding has also been professionalised and embedded into the planning and strategies of the City of Malmö. This is a sign of maturity, and could mean that the phenomenon is not considered deviant, disturbing or rebellious but, rather, natural. The question arises whether the maturation of the skateboarding-friendly approach in Malmö, in combination with the (aging) skateboarders being now established professionals in different organisations and positions, has undermined the edgy, alternative, saucy character of skateboarding. In Malmö, skateboarders do not have to (re)claim space and make noise to get what they want. In general, this is of course positive. However, there is a risk of a widening gap between a new generation of skaters and the older generation. Using a metaphor, the skateboarders in Malmö have been served LOVE Malmö on a silver platter. Value is created for the city as well as for the skate community, but maybe the need for co-creation has decreased as the processes have developed and become professionalised and integrated.

Also, as expressed by, for example, Borden (6), skateboarders sometimes act as gatekeepers to protect the codes of their culture, and the skateboarding community has historically been sceptical about advances from uninvested parties. The younger generation may not be aware that the facilitators of the spectacularly skate-friendly approach in Malmö, among others the skateboarding coordinator, are part of the skate community.

Furthermore, as mentioned in one of the interviews, the organisational structure of collaborations is complicated to sustain. Perhaps the success of the Malmö approach to skateboarding has created not only globally and locally celebrated values and inspiration for other cities, but also a gap in relation to the grassroots. It may be that new ways of going back to the grassroots, engaging the new generation and getting new ideas and input, will be found, as well as ways to avoid expectations without commitment from the skaters. In the interviews, those having the power in the skateboarding city Malmö stress the need to see and revitalise the grassroots. In the long run, creativity probably requires friction.

Malmö is doing most things right, but it could be time to involve and challenge new skateboarders to be innovative in the new landscape of skateboarding, between the growing professionalisation and formalisation and an increasing interest in vibrant public spaces and citizens’ participation. O'Connor et al. (52) discuss “how skateboarding demonstrates a greyness” in terms of “political and environmental ambiguities, contradictions, liminality, nuances and paradoxes” (p. 898). Maybe this greyness of skateboarding on the border between different spheres, spaces and geographies, creates good opportunities for taking placemaking and value co-creation to the next level.

The skateboarding approach of Malmö shows a large array of success factors in terms of placemaking and value co-creation, locally but also internationally. It also shows that with success comes potential weaknesses. Professionalised structures and extensive networks and collaborations are hard to sustain and maybe also hard to penetrate for a newcomer. When a phenomenon, in this case skateboarding, becomes natural in the corridors of power, it is no longer visible as a unique magnet for attention. Then you need to revive the attention. Re-building LOVE Park in the shape of LOVE Malmö might have been such an effort.

One positive aspect of skateboarding being integrated as a natural part in the urban development is the spill-over effects on other sectors and issues in the city. An approach of placemaking and value co-creation that can be used in connection to other issues or interests as well, has been developed around skateboarding in Malmö.

Finally, the case of Malmö shows the importance of not only integrating the community but also continuing to do so when the development is successful.

In future research, I plan to examine the new generation of skateboarders as part of the placemaking and value co-creation, in the skateboarding city where skateboarders do not need to be rebellious.

## Data Availability

Requests to access the datasets should be directed to karin.book@mau.se.
